# Endometriosis leads to central nervous system-wide glial activation in a mouse model of endometriosis

**DOI:** 10.1186/s12974-023-02713-0

**Published:** 2023-03-06

**Authors:** Shah Tauseef Bashir, Catherine R. Redden, Kishori Raj, Rachel B. Arcanjo, Sandra Stasiak, Quanxi Li, Andrew J. Steelman, Romana A. Nowak

**Affiliations:** 1 Department of Molecular and Integrative Physiology, University of Illinois at Urbana-Champaign, Urbana, IL 61801 USA; 2 Department of Animal Sciences, University of Illinois at Urbana-Champaign, 1207 W. Gregory Drive, Room 314 ASL, Urbana, IL 61801 USA; 3 Department of Comparative Biosciences, University of Illinois at Urbana-Champaign, Urbana, IL 61801 USA

**Keywords:** Endometriosis, Chronic pelvic pain (CPP), Hyperalgesia, Glial activation

## Abstract

**Background:**

Chronic pelvic pain (CPP) is a common symptom of endometriosis. Women with endometriosis are also at a high risk of suffering from anxiety, depression, and other psychological disorders. Recent studies indicate that endometriosis can affect the central nervous system (CNS). Changes in the functional activity of neurons, functional magnetic resonance imaging signals, and gene expression have been reported in the brains of rat and mouse models of endometriosis. The majority of the studies thus far have focused on neuronal changes, whereas changes in the glial cells in different brain regions have not been studied.

**Methods:**

Endometriosis was induced in female mice (45-day-old; *n* = 6–11/timepoint) by syngeneic transfer of donor uterine tissue into the peritoneal cavity of recipient animals. Brains, spines, and endometriotic lesions were collected for analysis at 4, 8, 16, and 32 days post-induction. Sham surgery mice were used as controls (*n* = 6/timepoint). The pain was assessed using behavioral tests. Using immunohistochemistry for microglia marker ionized calcium-binding adapter molecule-1 (IBA1) and machine learning “Weka trainable segmentation” plugin in Fiji, we evaluated the morphological changes in microglia in different brain regions. Changes in glial fibrillary acidic protein (GFAP) for astrocytes, tumor necrosis factor (TNF), and interleukin-6 (IL6) were also evaluated.

**Results:**

We observed an increase in microglial soma size in the cortex, hippocampus, thalamus, and hypothalamus of mice with endometriosis compared to sham controls on days 8, 16, and 32. The percentage of IBA1 and GFAP-positive area was increased in the cortex, hippocampus, thalamus, and hypothalamus in mice with endometriosis compared to sham controls on day 16. The number of microglia and astrocytes did not differ between endometriosis and sham control groups. We observed increased TNF and IL6 expression when expression levels from all brain regions were combined. Mice with endometriosis displayed reduced burrowing behavior and hyperalgesia in the abdomen and hind-paw.

**Conclusion:**

We believe this is the first report of central nervous system-wide glial activation in a mouse model of endometriosis. These results have significant implications for understanding chronic pain associated with endometriosis and other issues such as anxiety and depression in women with endometriosis.

**Graphical Abstract:**

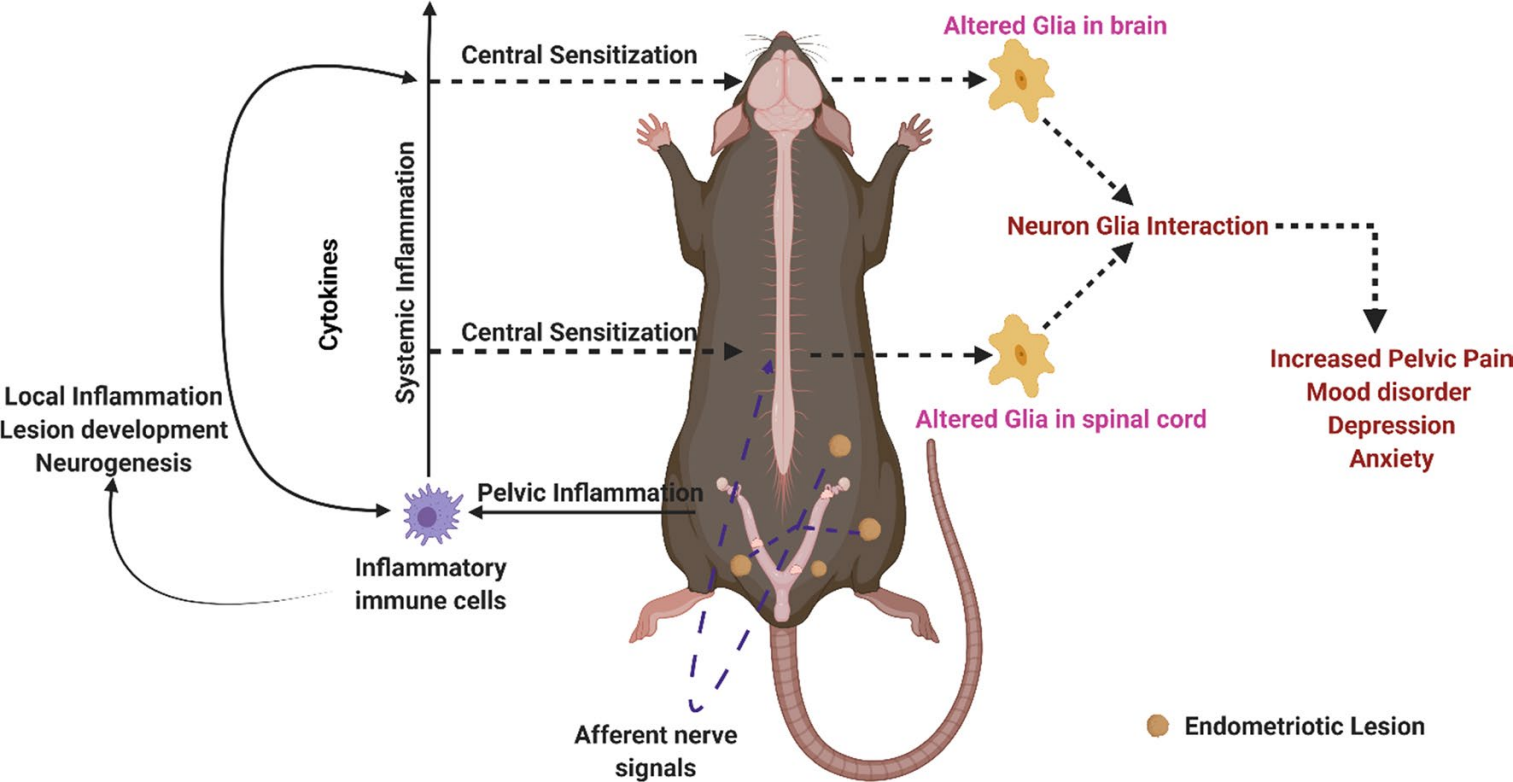

**Supplementary Information:**

The online version contains supplementary material available at 10.1186/s12974-023-02713-0.

## Introduction

Endometriosis is one of the most common reproductive disorders in women. Endometriosis is a chronic inflammatory disease where endometrial tissue grows on surfaces outside the uterus, which negatively impacts the reproductive system, causes chronic pain, and reduces the quality of life. The ectopic lesions are commonly discovered on the pelvic organs and peritoneum, but they can also be found on the ovaries, kidneys, intestines, bladder, skin, and lungs [1–3]. This gynecological disorder is widespread as it affects 5–10% of women of reproductive age [4–6]. Chronic pelvic pain (CPP) and infertility is observed in 30–50% of women with endometriosis [6, 7], which are the two most common symptoms of endometriosis [8]. Other symptoms of endometriosis include bowel pain, dysuria, dysmenorrhea [9], and menorrhagia [8]. Due to the chronic nature of the resulting pain, women with endometriosis report a significant decrease in quality of life and other mental health concerns such as anxiety and depression [10–13]. Additional endometriosis-associated comorbidities include irritable bowel syndrome and overactive bladder syndrome due to common innervation of the female reproductive tract, colon, and bladder [14]. The causes of CPP are complex and can be attributed to its multifaceted nature. Some of the factors contributing to CPP include inflammation associated with endometriotic lesions and the peritoneum [15, 16], activation of peripheral nerve endings [17, 18], and central sensitization [19–23].

Recent studies indicate a significant role of the immune system in endometriosis-associated pelvic pain. Classically activated (M1) macrophages are recruited to endometriotic lesions to secrete cytokines and may polarize to alternatively activated (M2) macrophages that mediate immune responses and stimulate the growth of nerves and blood vessels [24]. Disease-modified macrophages encourage the growth and activation of nerves by producing many neurotrophic factors such as nerve growth factor (NGF), insulin-like growth factor 1 (IGF-1), brain-derived neurotrophic factor (BDNF), and Neurotrophin-3 (NTF3) [25–29]. Previous studies have also shown that women diagnosed with endometriosis had higher concentrations of IGF-1 and NGF in the peritoneal fluid than women without endometriosis [30, 31]. Studies have also found the presence of increased nerve fiber density in and around endometriotic lesions in women with endometriosis and in endometriotic lesions in non-human primate, rat, and mouse models of endometriosis [17, 26, 32–37].

Although inflammation associated with endometriotic lesions, peritoneal inflammation, and activation of peripheral nerve endings explain some aspects of CPP, some studies have shown that CPP can persist or recur in women after the removal of endometriotic lesions [38]. In addition, the severity of pain experienced poorly correlates with the lesion load [39–41]. A potential mechanism to explain CPP is central sensitization, which involves long-lasting neuroplastic modifications in nociceptive neurons in the central nervous system (CNS). Persistent peripheral stimuli can alter neuronal circuits in nociceptive pathways, which cause heightened response to noxious stimuli (hyperalgesia) and pain response to innocuous stimuli (allodynia) [19, 42–46]. In addition to neurons, glial cells, especially microglia and astrocytes, are critical for developing central sensitization [47–50]. Changes in the functional activity of post-synaptic action potentials, functional magnetic resonance imaging signals, and gene expression have been reported in the brains of rat and mouse models of endometriosis [21, 51]. An increase in the expression of the glial fibrillary acidic protein (GFAP: an astrocyte marker) and the cluster of differentiation molecule 11B (CD11B: a microglia marker) has been reported in the dorsal horn of spinal cords in mice [52]. Moreover, pro-inflammatory cytokines released during the immune response to endometriotic lesions and signals from visceral afferents can affect glial cells leading to phenotypic changes or reactive gliosis. However, changes in the glial cells in different brain regions have not been studied in endometriosis. Overall, very little is known about the effect of endometriosis on the glial cells in the brain.

Women with endometriosis are at a high risk of suffering from anxiety, depression, and other psychological disorders. Women with endometriosis-associated CPP have higher rates of depression (~ 86%); even women with no endometriosis-associated pain report depressive symptoms at rates higher (38 vs. 4.8%) than those of healthy reproductive-age women [53, 54]. Glial cells are involved in depression and anxiety, with studies focusing more on the role of microglia in depression than the astrocytes [55, 56]. Some of the common ways in which microglia play a role in depression include activation of the NLRP3 inflammasome, purinergic receptor modulation, sterile inflammation, and changes in activation status in response to stress [57–59]. Moreover, women are more susceptible to neuro-immune changes in response to chronic stress leading to higher rates of depression and anxiety [60]. Therefore, considering the role of astrocytes and microglia in developing central sensitization, depression, and response to chronic stress due to endometriosis, it is important to evaluate the glial changes across different regions of the CNS caused by endometriosis.

In this study, we hypothesized that astrocyte and microglia phenotypes are altered in the brain in a mouse model of endometriosis during the early stages of endometriotic lesion establishment. We investigated the astrocytic and microglial phenotype changes using two previously reported markers [61–65] glial fibrillary acidic protein (GFAP) and ionized calcium-binding adapter molecule-1 (IBA1).

## Materials and methods

### Animals

Animal experiments were performed following approval from the University of Illinois Institutional Animal Care and Use Committee per the National Institutes of Health standards for the use and care of animals. Forty-five day old female C57/BL6 mice were purchased from Charles River (027C57BL/6) and housed in an environment-controlled animal facility (12:12 light–dark cycle) with ad libitum access to food and water.

### Induction of endometriosis

**Fig. 1:**
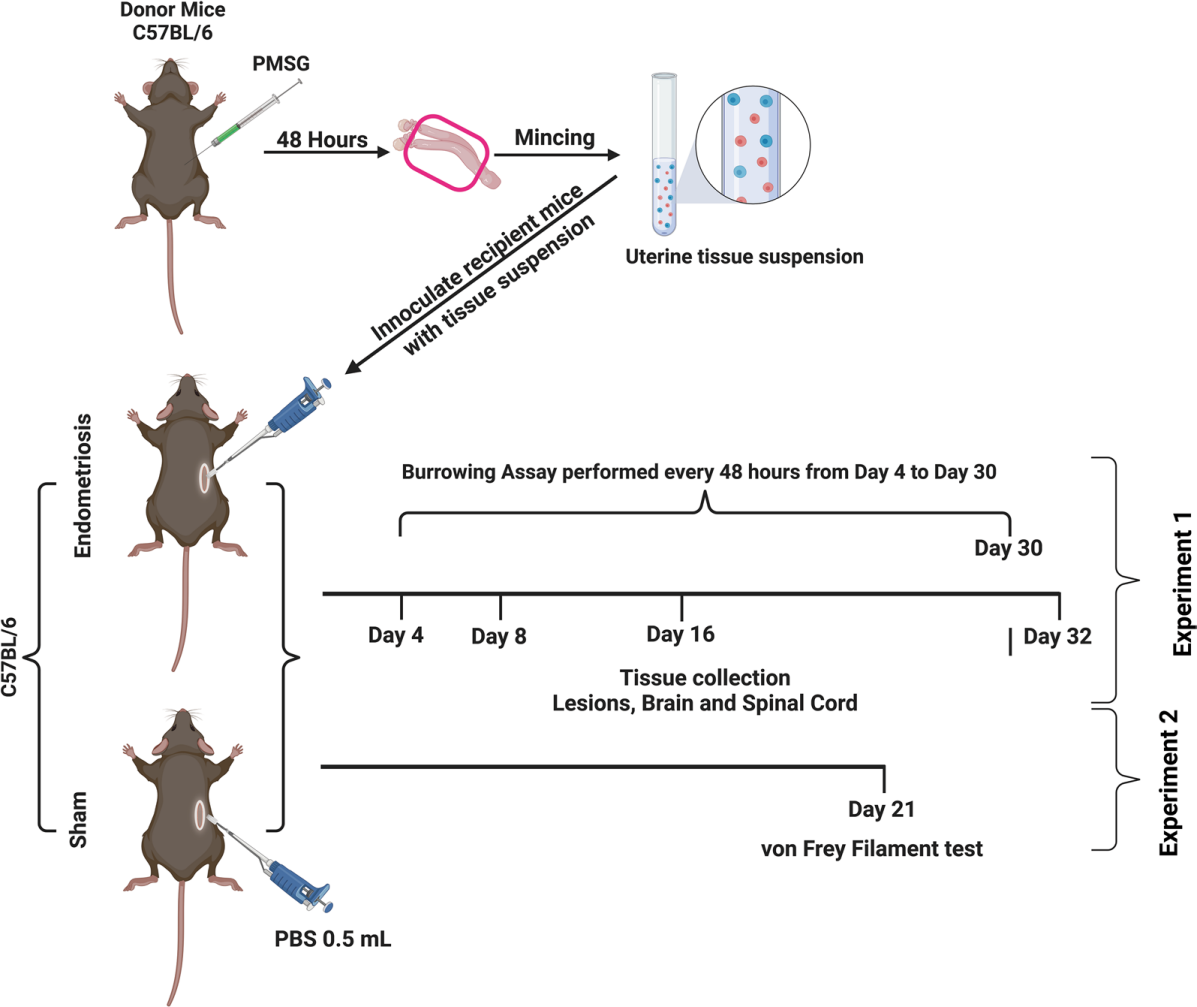
Experimental timeline (created with BioRender.com). Syngeneic female donor mice were primed with 5 IU of pregnant mare serum gonadotropin (PMSG; i.p.) to stimulate endometrial growth for 48 h before euthanasia. Immediately after euthanasia, uterine horns were isolated, minced (< 0.5 mm), washed with sterile phosphate-buffered saline (PBS), and resuspended in 1.0 ml of PBS to create a uterine tissue suspension. While recipient mice were anesthetized, a small dorsolateral incision (5 mm) was made, and 0.5 mL of tissue fragment suspension from a single uterine horn (Endometriosis group; experiment-1 *n* = 6–11/timepoint, experiment-2 *n* = 6) or sterile PBS only (Sham group; experiment-1 *n* = 6/timepoint, experiment-2 *n* = 6) was carefully injected into the peritoneal cavity of the recipient mice. Finally, the lesions, brains, and spinal cords were collected from recipient mice euthanized in estrus (based on vaginal smears) at ~ 4, ~ 8, ~ 16, and ~ 32 days after induction of endometriosis

Syngeneic female donor mice were primed with 5 IU of pregnant mare serum gonadotropin (PMSG; i.p.) to stimulate endometrial growth 48 h before euthanasia. Immediately after euthanasia, uterine horns were isolated and cut into small pieces (< 0.5 mm), washed with sterile phosphate-buffered saline (PBS), and resuspended in 1.0 ml of PBS. A similar model of endometriosis induction has previously been used in mice, albeit with estradiol supplementation [66]. Recipient mice were anesthetized using ketamine/xylazine (87 mg/kg; 15 mg/kg; i.p.). A small dorsolateral incision (5 mm) was made, and 0.5 ml of tissue fragment suspension containing uterine tissue equal to 1 uterine horn (Endometriosis group; experiment-1 *n* = 6–11/timepoint, experiment-2 *n* = 6) or sterile PBS only (Sham group; experiment-1 *n* = 6/timepoint, experiment-2 *n* = 6) was carefully injected into the peritoneal cavity of the recipient mice. Closure of the peritoneal cavity was achieved by simple interrupted suturing of the muscle layer with plain gut 5–0 and wound clips for skin incisions. Recipient mice also received buprenorphine (0.05 mg/kg: s.c.) 1 h before administering anesthesia and 6–10 h after the initial dose to reduce pain from surgery. Mice were euthanized in estrus (based on vaginal smears) at ~ 4, ~ 8, ~ 16, and ~ 32 days after induction of endometriosis (Fig. 1). Mice were transcardially perfused with sterile PBS, endometriotic lesions, brains, and spines were collected and placed in 10% neutral buffered formalin for 24 h, then transferred to 70% ethanol until further processing.

### Confirmation of endometriosis

Peritoneum and internal organs were thoroughly examined for the presence of endometriotic lesions under a dissection microscope. Suspected endometriotic lesions were collected and placed in 10% neutral buffered formalin for 24 h, followed by 70% ethanol. Gross lesions were stained with hematoxylin and eosin (H&E) to identify endometrial glands and stroma to confirm these were endometriotic lesions. Mice without endometriosis were excluded from further analyses.

### Burrowing assay

The burrowing assay measures changes in spontaneous behavior as an indirect assessment of pain/discomfort. The assay was performed as previously described [67–69] with minor modifications. Mice (*n* = 4/group) were individually housed and acclimatized to the burrowing tube for three days before the start of data collection with food pellets refilled into the tube every 24 h. Beginning on day 4, the burrowing tube was filled every other day with 125 g of the food pellets at 0 h (0 h) and placed into the cage. The remaining pellets in the tube were weighed at 20 min, 2 h (2 h), 3 h (3 h), 5 h (5 h), 7 h (7 h), and 24 h (24 h). The tube was refilled, and measurements were repeated every 48 h until day 30. Data from the burrowing assay was divided into two time periods, before day 15 (day 4 to 14) and after day 15 (day 16 to 30).

### Von Frey filament test

The Von Frey filament test is used to measure allodynia and hyperalgesia and is a more direct way to measure visceral pain and hyperalgesia. Mice were individually housed and acclimatized to the modular animal enclosures (Stoelting, 57823) at least 3 days before the experiment. Tests were performed using Semmes–Weinstein Von Frey filaments (Stoelting, 58011) as previously described [70, 71]. Filaments were applied to the abdomen or hind-paw ten times, the filament evoking a withdrawal response in at least 50% of applications was considered the threshold force (*g*).

### Immunohistochemistry

Spinal columns were decalcified using 0.5 M EDTA to soften them for further processing and sectioning. Subsequently, the brains and spinal cords were processed in a VipTek tissue processor and paraffin embedded. Tissues were sectioned into 8-μm-thick sections using a microtome and transferred to Superfrost Plus slides (Fisher Scientific). Immunohistochemical staining was performed at least 24 h after slides were allowed to dry.

Tissue sections were deparaffinized using three washes of xylene and rehydrated using a series of decreasing ethanol concentrations. Heat-induced antigen retrieval was performed with citrate buffer (pH 6) at 100 °C for 30 min, and slides were allowed to cool to room temperature (RT) in the buffer. Endogenous peroxidase activity was inactivated with 0.9% H_2_O_2_ for 20 min or Bloxall endogenous blocking solution (Vector Laboratories) for 10 min. The slides were then washed with phosphate-buffered saline with 0.1% Tween-20 (PBST) and incubated in a blocking solution consisting of 10% normal goat serum (Vector Laboratories) diluted in 1% BSA/PBST (0.5% Tween-20) at RT for 1 h. For tumor necrosis factor (TNF) immunohistochemistry, tissues were blocked with 10% normal goat serum in PBS. The tissue sections were then incubated with primary antibodies overnight at 4 °C in a humidified chamber. The primary antibodies used were: ionized calcium-binding adapter molecule-1 (rabbit anti-IBA1:1:2,000; Wako, 019–19,741), glial fibrillary acidic protein (chicken anti-GFAP; 1:5000; MiliporeSigma, AB5541), rabbit anti-interleukin (IL)-6 (1:250; Abcam, ab208113), rabbit anti-TNF (1:500; ThermoFisher, AMC3012), and rabbit anti-KI67 (1:2,000; Abcam, ab15580). Primary and secondary antibody controls were used to confirm the specificity of the primary/secondary antibodies when necessary, for negative control non-specific IgG was used and for secondary antibody control a “no primary antibody” control was used. The following day, slides were washed once with PBST for 10 min and twice with PBS for 5 min each. Next, slides were incubated with goat-anti-rabbit or goat-anti-chicken biotinylated secondary antibody (Vector Laboratories, BA-1000 and BA-9010, respectively) at 1:800 dilution in PBST (0.1% Tween-20) for 60 min at RT. Slides were then washed as in the previous step and incubated in ABC reagent (Vectastain ABC kit, Vector Laboratories) for 30 min at RT. After washing again, as in previous steps, slides were incubated with chromogen 3′3-diaminobenzidine (DAB; Vector Laboratories); PBS or water was used to stop the DAB reaction. Subsequently, the slides were counterstained with hematoxylin for 30 s, followed by dehydration, clearing, and cover-slipping. Coverslipped slides were dried for at least 24 h before scanning.

### Image analyses

Immunostained slides were scanned with the Hamamatsu NanoZoomer-XR scanner. Images were exported using NanoZoomer digital pathology software (NDPI) at 40× magnification (approximately 1 mm by 0.5 mm in size) from the cortex, hippocampus, thalamus, and hypothalamus. A blinded user exported images to reduce any experimental bias. Exported images were deconvoluted using the inbuilt color Deconvolution (H-DAB) function in Fiji image analysis software [72] to reduce image size. Deconvolution allowed faster processing to obtain “Brown” stained areas (positive immunoreaction). After that, images were loaded into the machine learning “Weka trainable segmentation” plugin in Fiji, and the plugin was trained to identify three classes of immunostaining: soma, filaments, and background. Once the machine learning algorithm was able to identify different classes with > 99.9% probability, images were further processed to create a probability map of the input image (Additional file 2: Fig. S1) [73]. Processed images were changed from 32-bit to 8-bit images and thresholded. Soma size was measured using “Analyze particle function” in Fiji with a size threshold of 30—infinity and circularity threshold of 0.2—infinity. For determining the percentage area (total cell size) analysis of IBA1 and GFAP, we calculated the total area under brown immunoreactivity divided by the total tissue area analyzed. For the relative expression of IL6 and TNF, the percentage area of brown immunoreactivity was calculated, and then the data from endometriotic mice were normalized to the values of sham control mice.

### Statistical analysis

All statistical analysis was done using GraphPad Prism 9.1.0. Data were tested for normal distribution using the Shapiro–Wilk normality test. If data were normally distributed, unpaired two-tailed *t*-test or ordinary one-way ANOVA with multiple comparisons with Dunn’s test were used. If data were not normally distributed, Mann–Whitney or Kruskal–Wallis test was performed, and multiple comparisons with Holm–Šídák's test. For burrowing data, multiple comparisons were done by controlling for false discovery rate (FDR) using a two-stage linear step-up procedure of Benjamini, Krieger, and Yekutieli as available in GraphPad Prism. Data are shown as mean ± SEM unless otherwise specified. A *p*-value < 0.05 was considered significant.

## Results

### Gross morphology and histology

**Fig. 2:**
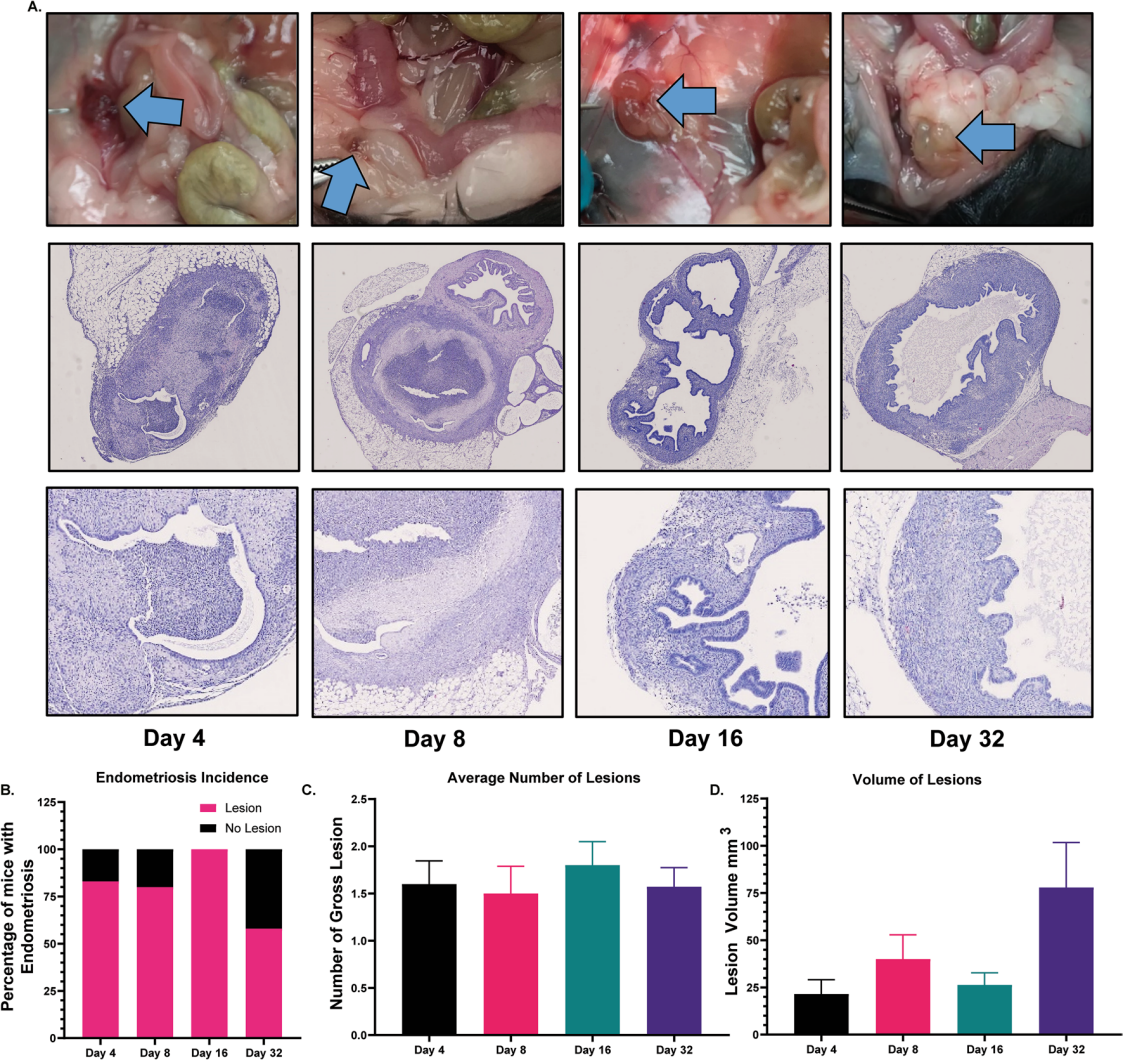
Confirmation of endometriotic lesions. **A** Gross endometriotic lesions are shown in the top row. The blue arrow points to the endometriotic lesions on days 4, 8, 16, and 32. The second and third rows show 5X and 40X magnified histological sections of the lesions. **B** The percentage of mice with endometriotic lesions was 83.3% (5/6), 80% (8/10), 100% (10/10), and 64% (7/11) on days 4, 6, 16, and 32, respectively. **C**, **D** No differences were observed in the average number of lesions and lesions volume on different days after induction of endometriosis

Endometriotic lesions were confirmed on days 4, 8, 16, and 32 through gross morphology and histology (Fig. 2A). The blue arrows in Fig. 2A point to endometriotic lesions from the gross perspective. Histological evaluation of the lesions shows the presence of uterine glands and stroma. The percentage of mice with endometriotic lesions was 83.3% (5/6), 80% (8/10), 100% (10/10), and 64% (7/11) on days 4, 8, 16, and 32, respectively (Fig. 2B). The average number of lesions and lesion volumes did not differ in mice euthanized at the different time points (Fig. 2C, D).

### Behavioral assessment

**Fig. 3:**
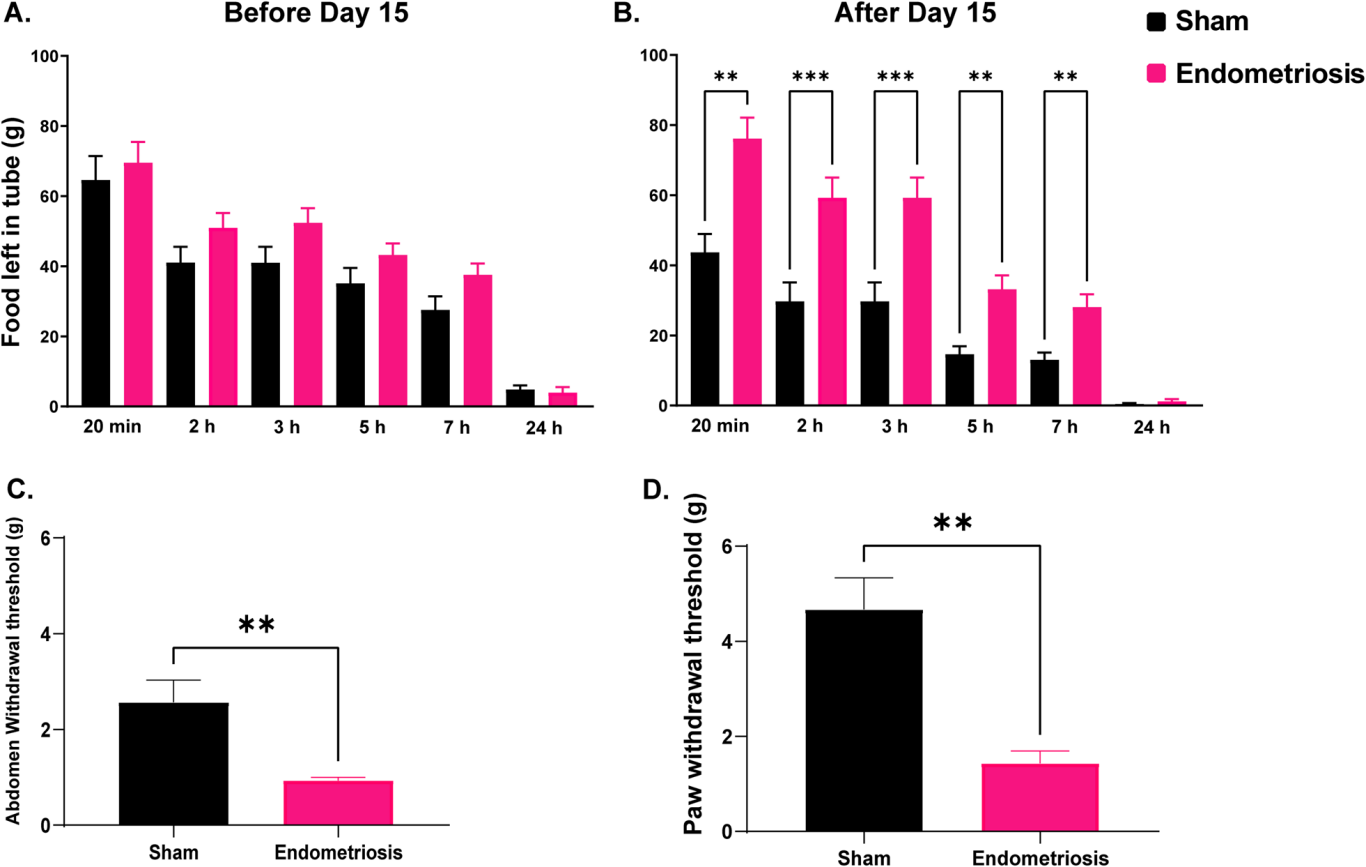
Burrowing and Von Frey assay. Burrowing activity in sham and endometriotic mice were assessed by measuring the weight of food left inside the tube at the following timepoints: 20 min, 2 h, 3 h, 5 h, 7 h, and 24 h. **A** No differences in burrowing behavior were observed before day 15 (days 4–14) between sham controls and endometriotic mice. **B** After day 15 (days 16–30), mice with endometriosis had reduced burrowing than sham controls at all timepoints except at 24 h. Von Frey assay showed decreased **C** abdominal withdrawal and **D** Paw withdrawal threshold in endometriotic mice. Values represent mean ± standard error mean (SEM), *n* = 4–6 mice/timepoint. The asterisks indicate significant differences between groups, **(*p* < 0.01) and ***(*p* < 0.001)

The burrowing assay was conducted to assess changes in spontaneous behavior reflecting increased pain and discomfort in mice with endometriosis. Burrowing behavior is inherent in healthy, normal mice; therefore, food pellets would typically be removed from the tube quite quickly (Additional file 1: Video S1). Mice with pain or discomfort show reduced burrowing behavior resulting in slower removal of food pellets from the tube. No significant differences in burrowing behavior were observed between sham and endometriotic groups before day 15. However, after day 15, endometriotic mice showed a significant reduction in burrowing activity compared to sham control mice at all but the 24-h timepoint (Fig. 3A, B). Von Frey testing revealed increased abdominal (Fig. 3C) and hind-paw (Fig. 3D) hyperalgesia in endometriotic mice compared to control mice.

### Immunohistochemistry of glial and immune markers in various regions of the brain

To evaluate microglial activation, we measured the size of the microglial cell body (soma size). Activated microglia have been shown to have larger soma than resting microglia, and this can be measured by IBA1 immunostaining. Moreover, an altered number of microglia can also indicate neuroinflammatory changes in the brain. Therefore, soma size, IBA1 expression, and the number of microglia were assessed in the cortex, hippocampus, thalamus, and hypothalamus on day 4, day 8, day 16, and day 32 after tissue inoculation. These regions were selected due to their role in pain processing (prefrontal cortex); pain memory, depression, and anxiety (hippocampus); pain modulation and relaying signals (thalamus); and mood disorders, stress control, and reproductive function (hypothalamus).

**Fig. 4:**
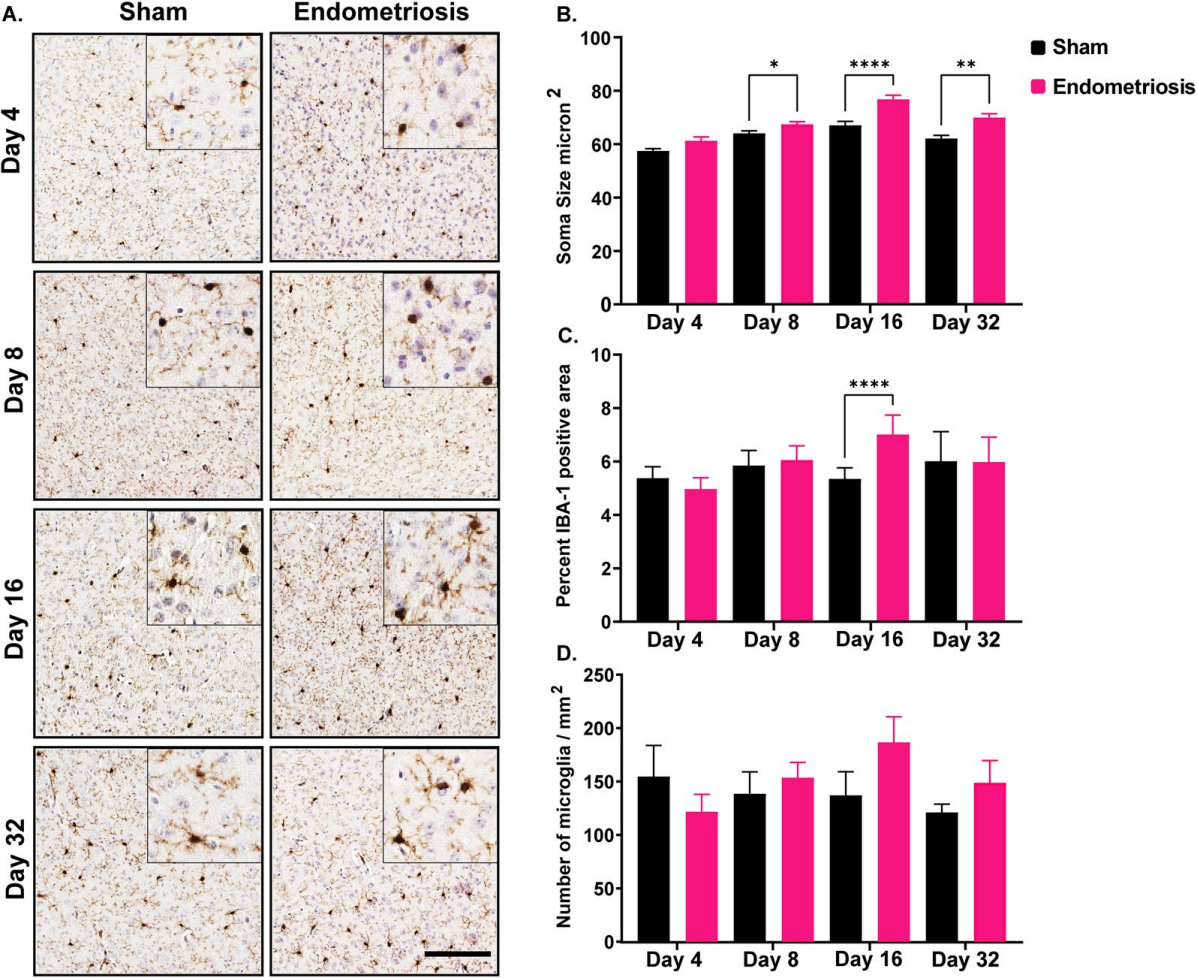
Microglial measurements in the cortex of sham controls and endometriotic mice. **A** Representative immunohistochemistry images of IBA1 in sham controls and endometriotic mice at various timepoints, the black bar in the lower right image is equal to 150 and 50 µm for the images and the insets, respectively. **B** Endometriotic mice had larger soma size on average than sham controls on days 8, 16, and 32. **C** Endometriotic mice also had increased IBA1 expression on day 16. **D** No difference in the microglia numbers was observed between the sham control and endometriotic mice. Values represent mean ± standard error mean (SEM), *n* = 5–6 mice/timepoint. The asterisks indicate significant differences between groups, *(*p* < 0.05), **(*p* < 0.01), and ****(*p* < 0.0001)

In the cortex, the microglia of endometriotic mice had significantly larger somas on days 8, 16, and 32 compared to sham controls (Fig. 4). Soma sizes in endometriotic and sham mice were not significantly different on day 4. In addition to changes in the soma size, mice with endometriosis showed a significantly increased area with IBA1 expression at day 16 compared to shams (Fig. 4). IBA1 expression was not different between sham and endometriotic mice on days 4, 8, and 32. Finally, the number of microglia was not significantly altered between sham and endometriotic mice across the different time points (days 4, 8, 16, and 32).

**Fig. 5:**
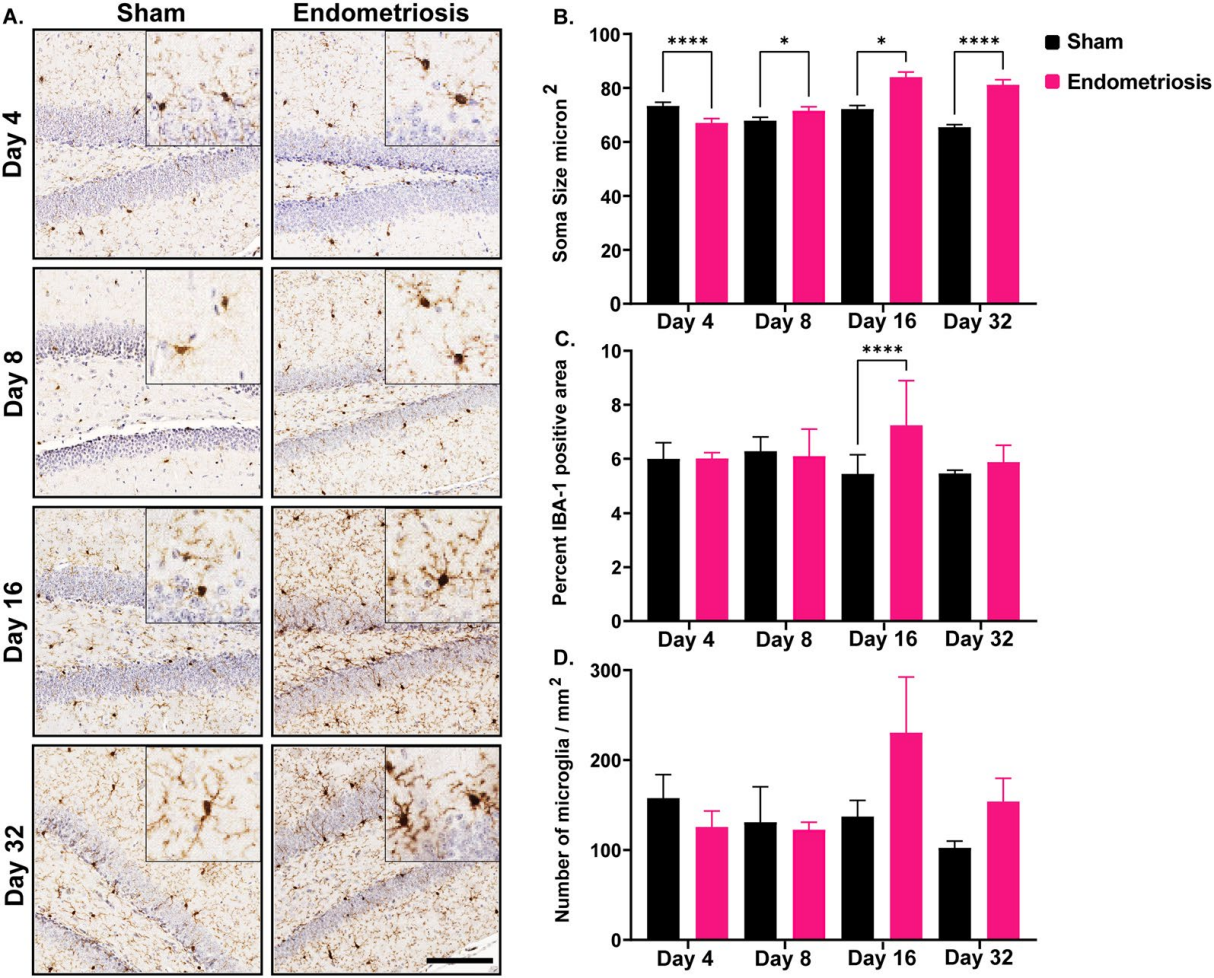
Microglial measurements in the hippocampus of sham controls and endometriotic mice. **A** Representative immunohistochemistry images of IBA1 in sham controls and endometriotic mice at various timepoints, the black bar in the lower right image is equal to 150 and 50 µm for the images and the insets, respectively. **B** Endometriotic mice had larger soma size on average than sham controls on days 8, 16, and 32. However, the soma size of the endometriotic mice was smaller on day 4 than sham controls. **C** Endometriotic mice also had increased IBA1 expression on day 16. **D** No difference in the microglia numbers was observed between the sham control and endometriotic mice. Values represent mean ± standard error mean (SEM), *n* = 5–6 mice/timepoint. The asterisks indicate significant differences between groups, *(*p* < 0.05) and ****(*p* < 0.0001)

We next assessed soma size, IBA1 expression, and the number of microglia in the hippocampus. In the hippocampus, the microglia of mice with endometriosis had significantly different soma sizes compared to sham controls at all time points. While soma size in endometriotic mice was significantly smaller on day 4 compared to shams (Fig. 5), mice with endometriosis had significantly larger soma size than shams on days 8, 16, and 32. Similar to the cortex, endometriotic mice had significantly increased IBA1-positive area in the hippocampus at day 16 compared to shams (Fig. 5). IBA1 expression in the hippocampus was not significantly different between endometriotic and sham control mice on days 4, 8, and 32. Similar to the cortex, the number of microglia in the hippocampus was not significantly different between sham and endometriotic mice at any time point.

**Fig. 6:**
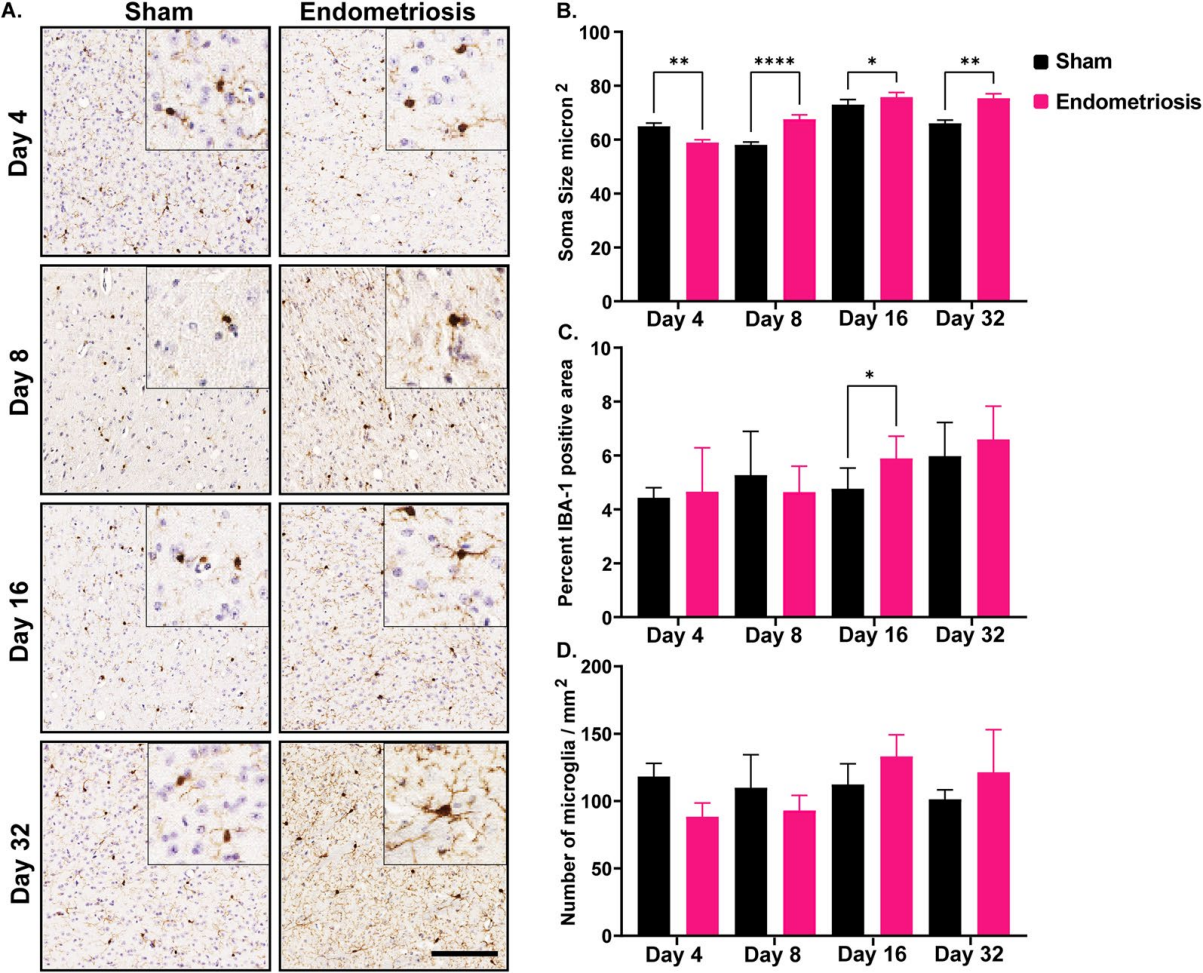
Microglial measurements in the thalamus of sham and endometriotic mice. **A** Representative immunohistochemistry images of IBA1 in sham controls and endometriotic mice at various timepoints, the black bar in the lower right image is equal to 150 and 50 µm for the images and the insets, respectively. **B** Endometriotic mice had larger soma size on average than sham controls on days 8, 16, and 32. However, the soma size of the endometriotic mice was smaller on day 4 than sham controls. **C** Endometriotic mice also had increased IBA1 expression on day 16. **D** No difference in the microglia numbers was observed between the sham control and endometriotic mice. Values represent mean ± standard error mean (SEM), *n* = 5–6 mice/timepoint. The asterisks indicate significant differences between groups, *(*p* < 0.05), **(*p* < 0.01), and ****(*p* < 0.0001)

Following the evaluation of the hippocampus, soma size, IBA1 expression, and the number of microglia were assessed in the thalamus. Endometriotic mice had significantly smaller soma size on day 4 compared to sham controls (Fig. 6), but had significantly larger somas on days 8, 16, and 32 than sham controls. The percent of the area with IBA1 expression in endometriotic mice was also significantly increased on day 16 compared to shams (Fig. 6), but was not significantly different on days 4, 8, and 32. Finally, the number of microglia in sham and endometriotic mice was not significantly altered across timepoints.

**Fig. 7:**
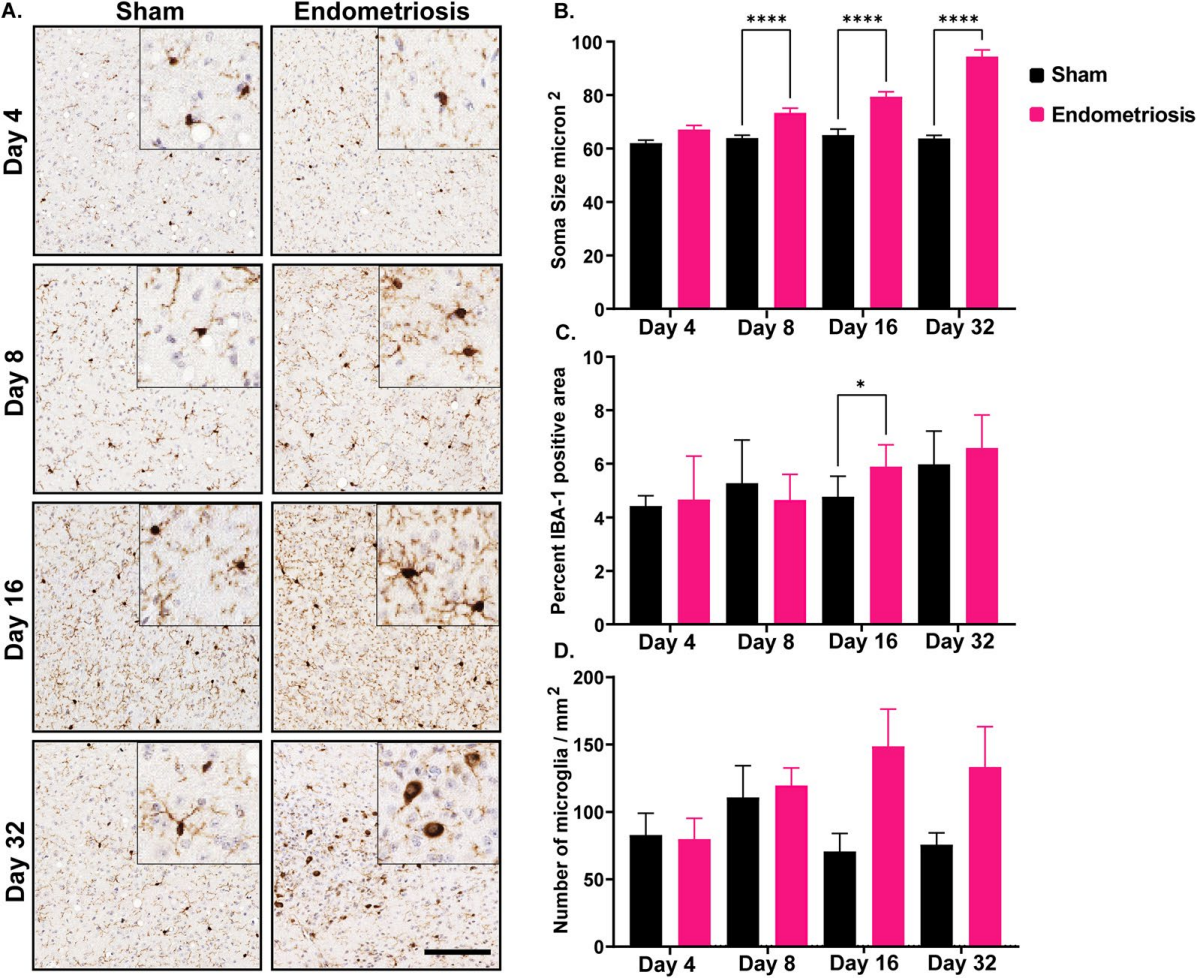
Microglial measurements in the hypothalamus of sham and endometriotic mice. **A** Representative immunohistochemistry images of IBA1 in sham controls and endometriotic mice at various timepoints, the black bar in the lower right image is equal to 150 and 50 µm for the images and the insets, respectively. **B** Endometriotic mice had larger soma size on average than sham controls on days 8, 16, and 32. **C** Endometriotic mice also had increased IBA1 expression on day 16. **D** No difference in the microglia numbers was observed between the sham control and endometriotic mice. Values represent mean ± standard error mean (SEM), *n* = 5–6 mice/timepoint. The asterisks indicate significant differences between groups, *(*p* < 0.05) and ****(*p* < 0.0001)

Lastly, the hypothalamus showed similar changes to those observed in the cortex. Mice with endometriosis had significantly larger somas in the hypothalamus on days 8, 16, and 32 compared to sham mice but not on day 4 (Fig. 7). Mice with endometriosis also displayed significantly increased area with IBA1 expression at day 16 compared to shams (Fig. 7). However, there were no differences in IBA1 expression on days 4, 8, and 32 between the sham control and endometriotic mice. The numbers of microglia in the hypothalamus of sham and endometriotic mice were not significantly different at any time point.

**Fig. 8:**
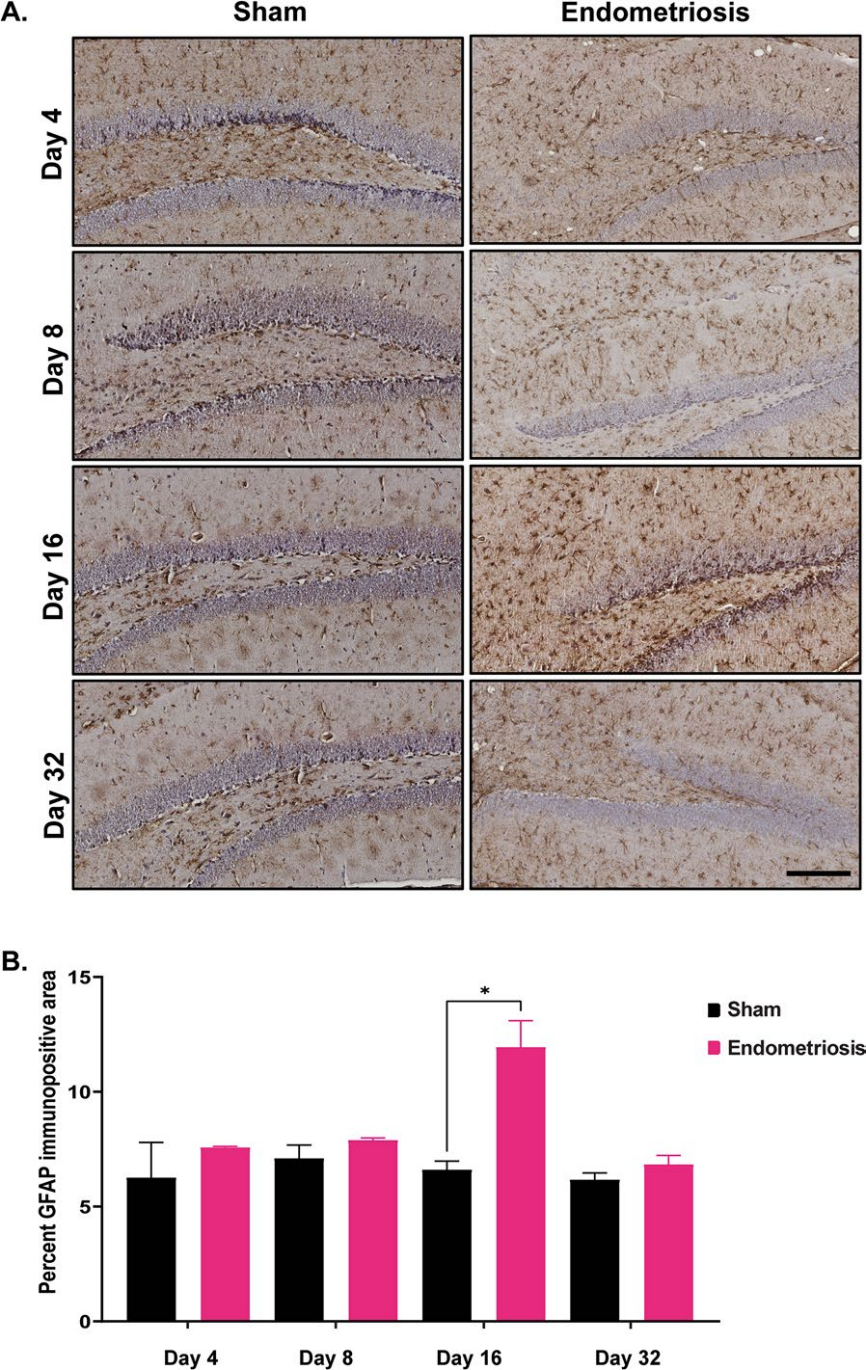
GFAP immunostaining in the hippocampus of sham and endometriotic mice. **A** Representative immunohistochemistry images of GFAP in sham controls and endometriotic mice on days 4, 8, 16, and 32, the black bar in the lower right image is equal to 150 µm. **B** Endometriotic mice had increased GFAP expression than sham controls on day 16. No differences were observed on any other days. Values represent mean ± standard error mean (SEM), *n* = 5–6 mice/timepoint. The asterisks indicate significant differences between groups, *(*p* < 0.05)

In addition to quantifying microglia, astrocytes were also analyzed using two markers, GFAP and S100 beta. GFAP immunostaining was performed on the hippocampus of sham control and endometriotic mice on days 4, 8, 16, and 32. On day 16, mice with endometriosis showed significantly more GFAP expression in the hippocampus than shams (Fig. 8). No significant differences in GFAP immunostaining were observed between the sham control and endometriotic mice on days 4, 8, and 32. Other brain regions, such as the cortex, thalamus, and hypothalamus, did not show consistent GFAP expression and therefore were not analyzed further (data are not shown). A second marker for astrocytes, S100-beta, was used to count the number of astrocytes in the cortex, hippocampus, thalamus, and hypothalamus of sham and endometriotic mice on days 16 and 32. The number of astrocytes was not significantly different between sham and endometriotic mice in any brain regions and across all time points (Additional file 2: Fig. S2).

Interestingly, glial heterogeneity was detected across several brain regions in sham control mice. Both the cortex and hippocampus contained significantly more microglia than the hypothalamus of sham mice. (Additional file 2: Fig. S3A). Soma size in the cortex, thalamus, and hypothalamus was significantly smaller than in the hippocampus of sham mice (Additional file 2: Fig. S3B). In addition, the number of astrocytes in the thalamus and hypothalamus were significantly greater than the number of astrocytes in the cortex of sham mice (Additional file 2: Fig. S3C). Endometriotic mice also showed glial heterogeneity across brain regions. Specifically, the number of microglia in the cortex was significantly higher compared to the thalamus (Additional file 2: Fig. S4A), and the number of astrocytes in the thalamus and hypothalamus were significantly increased compared to the hippocampus (Additional file 2: Fig. S4B).

**Fig. 9:**
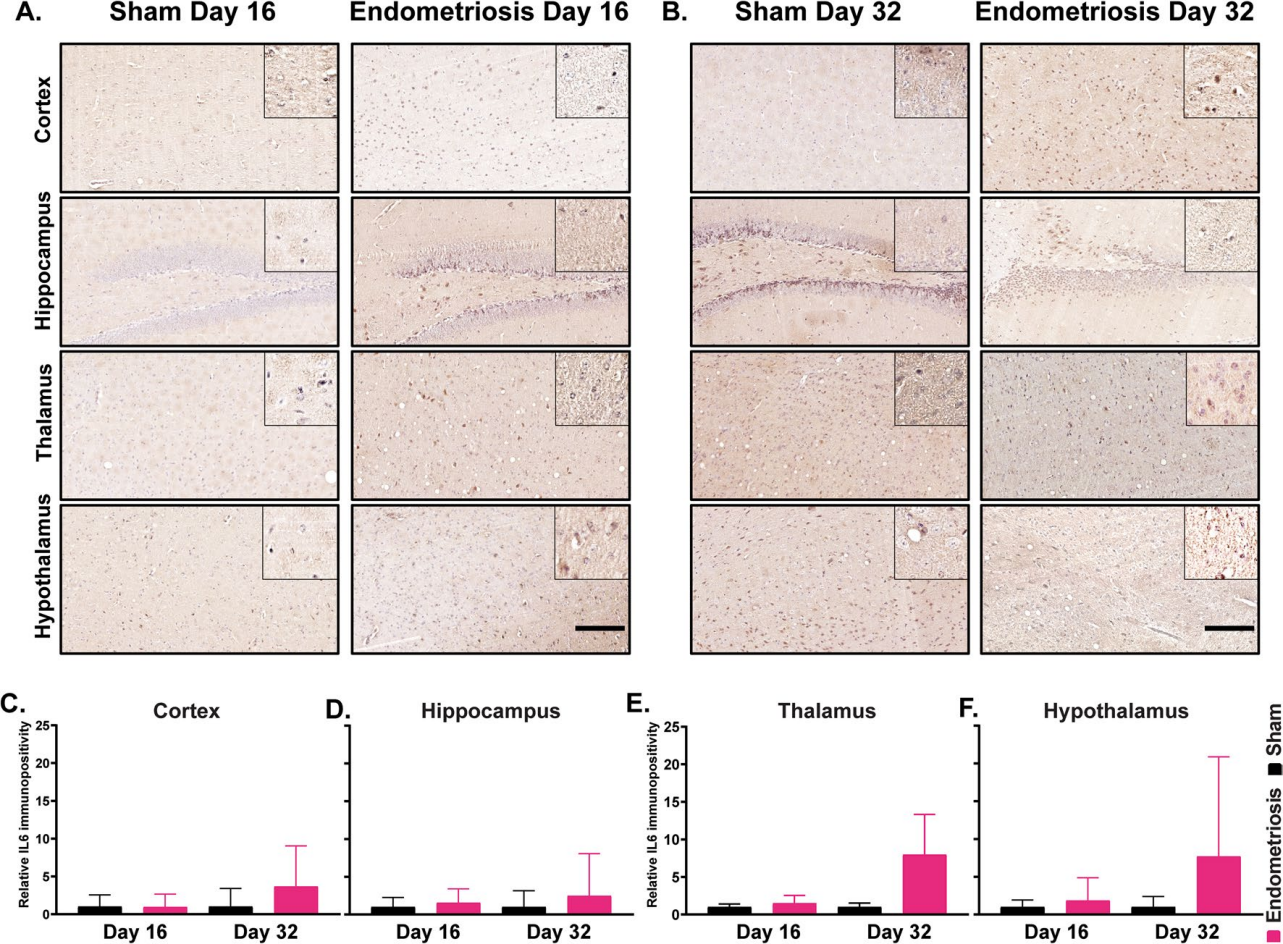
Immunostaining of IL6 in various brain regions of sham and endometriotic mice. **A** Representative immunohistochemistry images of IL6 in sham controls and endometriotic mice on day 16 and **B** day 32, the black bar in the lower right image is equal to 150 and 50 µm for the images and the insets, respectively. **C**–**F** No difference in IL6 expression was observed in the cortex, hippocampus, thalamus, and hypothalamus on days 16 and 32. Values represent mean ± standard error mean (SEM), *n* = 5–6 mice/timepoint

**Fig. 10:**
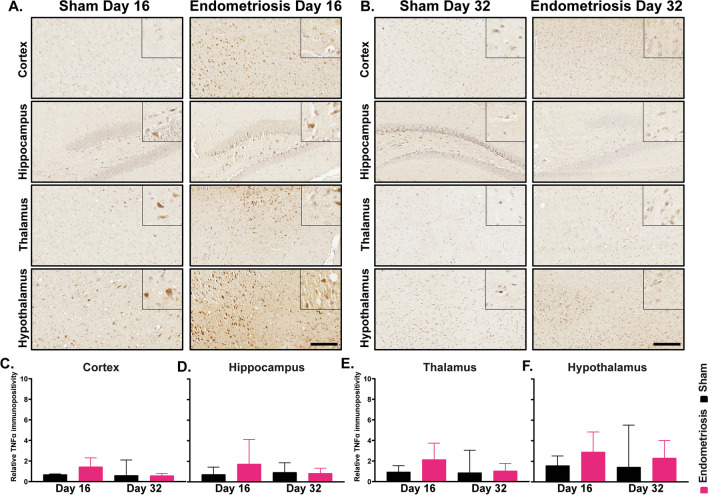
Immunostaining of TNF in various brain regions of sham and endometriotic mice. **A** Representative immunohistochemistry images of TNF in sham controls and endometriotic mice on day 16 and **B** day 32, the black bar in the lower right image is equal to 150 and 50 µm for the images and the insets, respectively. **C**–**F** No difference in TNF expression was observed in the cortex, hippocampus, thalamus, and hypothalamus on days 16 and 32. Values represent mean ± standard error mean (SEM), *n* = 5–6 mice/timepoint

Following immunostaining for the various glial markers, we next examined cytokine expression in various regions of the brain. Immunostaining was performed for IL6 and TNF in sham control and endometriotic mice in the various brain regions (cortex, hippocampus, thalamus, and hypothalamus) and timepoints (days 16 and 32). No differences were detected in IL6 expression in sham and endometriotic mice in any individual brain region or timepoints (Fig. 9). Similarly, no differences were detected in TNF levels for sham and endometriotic mice in any individual brain region or at any timepoint (Fig. 10). However, combining results for the individual regions revealed significant differences in expression between endometriotic and sham mice. Mice with endometriosis had significantly higher levels of IL6 on day 32 compared to shams; no differences were observed on day 16 (Additional file 2: Fig. S5A). In contrast, mice with endometriosis had significantly higher levels of TNF in the brain (cortex, hippocampus, thalamus, and hypothalamus combined) compared to shams; no differences were detected on day 32 (Additional file 2: Fig. S5B).

**Fig. 11:**
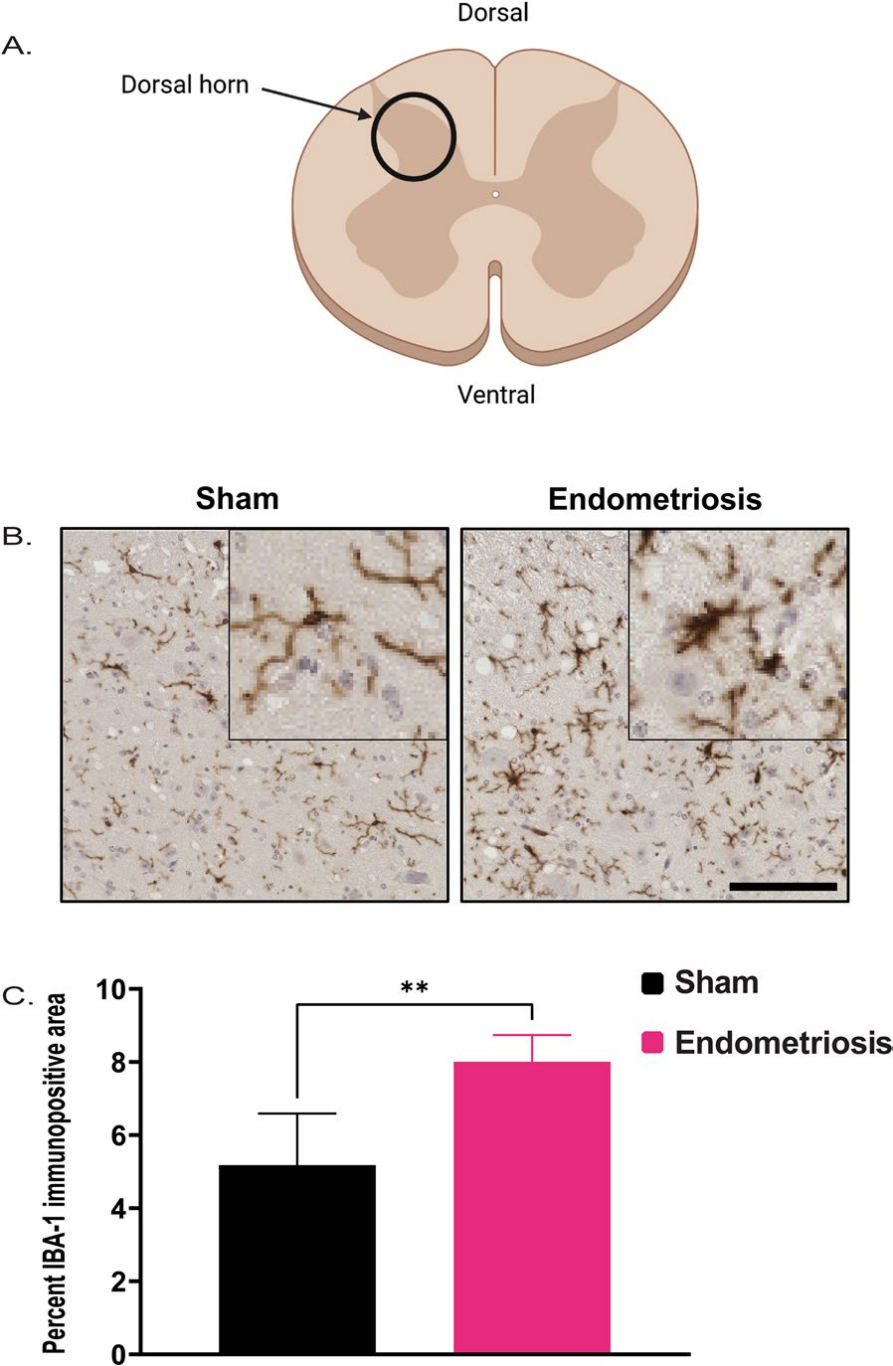
IBA1 Immunostaining in the spinal cord of sham and endometriotic mice. **A** Schematic diagram of the spinal cord (transverse section) indicating the location of the dorsal horn. **B** Representative immunohistochemistry images of IBA1 from the spinal cord in sham controls and endometriotic mice, the black bar in the lower right image is equal to 75 and 25 µm for the images and the insets, respectively. **C** IBA1 expression was increased in the spinal cords of mice with endometriosis than in sham controls. Values represent mean ± standard error mean (SEM), *n* = 7–12 mice. The asterisks indicate significant differences between groups, **(*p* < 0.01)

Lastly, the spinal cords of sham control and endometriotic mice were analyzed for IBA1 expression. Spinal cords from days 16 and 32 were combined for analysis. Mice with endometriosis showed significantly increased expression of IBA1 in the spinal cord compared to shams at the level of T13–L1 (Fig. 11).

## Discussion

Endometriosis is a complex multifactorial disease that affects women of reproductive age. The chronic and complex nature of endometriosis is further impacted by the large spectrum of symptoms leading to increased long-term healthcare costs. In addition to the concern of infertility, the occurrence of CPP, bowel pain, dysuria, dysmenorrhea [9], and menorrhagia [8] can severely impact patients with endometriosis. Recent research and meta-analysis studies have shown that women with endometriosis have a reduced quality of life and are at a higher risk of suffering from mental health disorders such as anxiety, depression, and other psychological ailments. Other factors that affect the quality of life in endometriosis patients include day-to-day concerns with fatigue and reduced work performance [11–13, 53].

Our results demonstrate CNS-wide glial activation in endometriotic mice. Specifically, we observed increased soma size in the cortex, hippocampus, thalamus, and hypothalamus. Increased soma size was observed 8 days after induction of endometriosis and persisted until the end of the experiment (day 32). Additionally, an increase in the IBA1-positive area was also observed on day 16 in all regions of the brain as well as the spinal cord at the thoracolumbar junction. Furthermore, we also observed an increased GFAP-positive area in the hippocampus. In combination with the burrowing behavior data, we show that the time course of glial activation correlates with the development of pain/discomfort in our model of endometriosis. While we did not observe differences in TNF and IL6 expression in individual brain regions, we did nevertheless observe increased TNF expression on day 16 and IL6 expression on day 32 when data from all brain areas were combined. The TNF and IL6 data require a more thorough analysis but, in general, support the histological observation of glial activation during endometriosis.

To our knowledge, this is the first study to report CNS-wide morphological changes in microglia in an animal model of endometriosis. These data are a step forward in better understanding the effects of endometriosis on the central nervous system and can help explain chronic pelvic pain due to central sensitization and a higher risk of mental health disorders. A small number of studies thus far have focused on the effects of endometriosis on CNS and subsequent implications in chronic pain, anxiety, and depression. Results from a recent study have shown hyperalgesia, altered post-synaptic transmission in glutamatergic and GABAergic synapses in the amygdala, altered gene expression, and anxiety and depression-like behavior in mice with endometriosis [51]. Additional studies have demonstrated altered functional magnetic resonance imaging (fMRI) changes as well as increased transient receptor potential vanilloid 1 (TRPV-1) and N-methyl-D-aspartate receptor (NMDAR) in neurons [22, 23]. Another study demonstrated that inhibition of fractalkine signaling in the spinal cord suppresses hyperalgesia and allodynia in rats with endometriosis [74]. Regarding studies on glial cells, one study has shown that endometriosis increases GFAP and CD11b expression in the spinal cord of mice with endometriosis [52]. One more study has shown increased GFAP and IBA1 expression in hippocampal lysates [75].

The exact mechanism by which CNS effects are observed in endometriosis are not fully understood. One possible mechanism includes systemic pro-inflammatory cytokines produced by the peritoneal macrophages crossing the blood–brain barrier (BBB). Another possible mechanism includes repeated afferent nerve signaling from the endometriotic lesion and peritoneum to the CNS, especially the dorsal root ganglion. There is ample evidence from studies in women and animal models demonstrating increased pro-inflammatory cytokines in the peritoneal fluid and serum due to endometriosis. Some of the cytokines such as IL6, TNF, IL-1β, VEGF, PGE2, IGF-1, NGF, BDNF, and NT-3 can act on the neurites locally as well as in the spinal cord leading to inflammatory and neurogenic effects locally and in the CNS [25–29, 34, 66, 70, 76–78].

Microglial activation is a broad term used to indicate a change in the physiological status of microglia from a normal quiescent state (resting) to a more activated state in response to external stimuli. Quiescent microglia are characterized by ramified processes and a small cell body (soma); upon activation, morphological changes can be observed in the form of larger soma size and shortening of the cellular processes [79]. Microglial activation is generally pro-inflammatory, but alternate anti-inflammatory forms are also observed [80]. Changes in microglial morphology such as increased soma size signify microglial activation and are highly correlated with altered cytokine levels [62, 63]. Our results showed that the microglia in the cortex of endometriotic mice had significantly larger somas on days 8, 16, and 32 after induction of endometriosis, indicating transformation from resting to an activated form. Studies have shown increased microglia activation in the mouse cortex after chronic constriction injury [81] or in animals exposed to chronic stress [82, 83]. Changes in cortical microglia are also associated with anxiety-like behavior and anhedonia [84]. Mice with endometriosis in our study showed reduced burrowing behavior indicative of behavioral dysfunction and discomfort due to pain.

Hippocampal and thalamic microglia soma size was also increased on days 8, 16, and 32 after induction of endometriosis, indicating activated microglia. Activation of hippocampal microglia is associated with chronic pelvic pain, chronic constriction injury, and depression-like behavior [81, 85, 86]. Moreover, hippocampal microglia activation can play a role in the sex differences observed in depressive behavior, predominantly affecting women [87]. Women are also more likely to develop central sensitization leading to hyperalgesia [88, 89] and are more likely to be affected by neuro-immune mediated anxiety and depression [60]. Similar to hippocampal microglia activation, thalamic microglia are activated through systemic signals from injuries, chronic pelvic pain, and stress [85, 90, 91]. Our results suggest that disruption of microglia due to the systemic effects of endometriosis can lead to central sensitization triggered chronic pain and increased predisposition to anxiety and depression. Changes in hippocampal and thalamic microglia soma size in the sham group on Day 4 can be ascribed to the microglial changes observed perioperatively as previously described [92].

Microglial soma size was also increased in the hypothalamus on days 8, 16, and 32 after induction of endometriosis. Microglia are involved in homeostasis by regulating the hypothalamus–pituitary–adrenal axis. Acute and chronic stress can activate microglia in the hypothalamus, but the effect of microglial activation on stress is not well understood [83, 91]. Hypothalamic inflammation has been implicated in dysregulation of the hypothalamus–pituitary–adrenal axis, triggering increased cortisol levels leading to cognitive and mood disorders [93]. More indications are emerging on the role of hypothalamic neuroinflammation on somatic and depressive disorders [94]. A recent study has shown that increased activation of hypocretin neurons in the hypothalamus of mice with endometriosis can modulate feeding behavior [95]. However, the specific role of hypothalamic microglial activation on mood disorders and other neuronal functions in the hypothalamus is not clear.

We observed reduced burrowing behavior in mice with endometriosis after day 15. Recovery from the original surgery can explain the lack of difference in burrowing activity before day 15 in sham and endometriotic mice. However, it is evident from our data that endometriosis has a marked effect on burrowing behavior after day 15. Furthermore, we observed increased abdominal and hind-paw hyperalgesia in endometriotic mice. In line with these finding, previous studies have reported increased hyperalgesia, allodynia, and pain behaviors in mouse and rat models of endometriosis. Some of the methods used to assess pain include abdominal-directed grooming, tunnel entry, mechanical sensitivity test (Von Frey filaments), and hot plate latency test. Our data are in agreement with the previously published reports of pain behaviors in mouse and rat models of endometriosis. In previous mouse studies, increased allodynia and pain behaviors were observed between 3–4 weeks after induction of endometriosis [25, 70]. A more recent study has shown that mechanical sensitivity to Von Frey filaments is increased even at 40–42 days after induction of endometriosis [96]. In transplantation models of endometriosis in rats, increased pain behaviors have been observed at 2, 4, 8, and 12 weeks after induction [21, 51, 75].

We also observed a significant increase in the IBA1 immunopositive area, an indicator of the total cell size of microglia (soma and processes), in mice with endometriosis on day 16 post-induction. Since enlarged soma and smaller processes are better indicators of microglial activation, we expected the total microglia area to differ from the soma size data. It is important to note that morphometric changes like soma size are gaining acceptance as an alternative to identify microglial activation and are associated with a better correlation with neuroinflammatory changes [62, 63, 65]. We also saw increased IBA1 immunopositive area in the spinal cord at the T13–L1 region in mice with endometriosis. Similar results have been reported with another microglia marker (CD11b) in a minimally invasive mouse model of endometriosis [52].

Also, on day 16, GFAP immunopositive area (an indicator for astrocyte cell size) was increased in the hippocampus of endometriotic mice, but no differences were observed on any other days. Increased total cell size in astrocytes is an indicator of astrocytic activation or reactive astrocytes [61]. Astrocytes play an important role in chronic pain, depression, and anxiety [47, 49, 97, 98]. Astrocytes and microglia interact and modulate each other's functions [99]. Astrocytes have also been shown to potentiate TNF secretion from microglia [100].

Finally, we observed no difference in TNF and IL6 expression in different brain regions individually but increases in both cytokines were observed when data from the brain as a whole were considered. An earlier study has shown increased *Tnf* expression in the spinal cord but not in the brains of mice with endometriosis [25]. In a prostatitis mouse model of chronic pelvic pain, increased levels of IL6 were observed in the thalamus and cortex but not in the hippocampus [85]. TNF and IL6 play an important role in central sensitization by modulating the post-synaptic terminal of both excitatory and inhibitory neurons [41]. Moreover, TNF and IL6 in the brain are also implicated in depression and anxiety-like behavior in mice [82, 86, 101, 102]. Therefore, more careful studies need to be done to determine the role of TNF and IL6 in endometriosis-associated central sensitization, anxiety, and depression.

Our study provides a valuable insight into the glial changes in a mouse model of endometriosis and help us to better understand the relevant biological mechanisms. However, it is important to keep in mind that there are differences between mice and humans and that the results obtained from our model may not necessarily translate directly to humans. As a result, it is important to carefully interpret the results of this study and to consider the potential for differences between species, when evaluating the relevance of the findings to humans.

## Conclusion

This study has provided compelling evidence of glial activation across brain regions in endometriosis. Microglia soma size was increased on days 8, 16, and 32 in endometriotic mice in the cortex, hippocampus, thalamus, and hypothalamus; these regions play an essential role in pain processing and mood, stress response, depression, and anxiety. Additionally, an increased percentage of GFAP immunoreactivity (indicated total astrocyte area) in the hippocampus was observed, which indicates activation of astrocytes. The majority of earlier studies have reported increased pain behaviors and other CNS effects 2 weeks after induction of endometriosis which is further corroborated by our study. Based on a plethora of evidence on the critical role of glial cells in pain, anxiety, and depression, it is likely that glial cells are involved in endometriosis-associated pain and other mental health disorder. It is important to determine if the microglia changes persist beyond day 32 or are transitory in nature: therefore, longer duration studies are required to scrutinize the long-term glial changes and their potential role in endometriosis-associated CPP, central sensitization, and possibly depression and anxiety. Studies to explore the mechanistic basis of glial activation in endometriosis may help identify potential targets for therapeutic interventions that can ameliorate the constant burden of pain and suffering and can considerably improve the quality of life in women with endometriosis.

## Supplementary Information

**Additional file 1.** Burrowing behavior in mice.

**Additional file 2.** Additional figures.

## Data Availability

The images used and analyzed during the current study are available from the corresponding author on reasonable request.

## Abbreviations

ANOVA: Analysis of variance
BBB: Blood brain barrier
BDNF: Brain-derived neurotrophic factor
BSA: Bovine serum albumin
CD11B: Cluster of differentiation molecule 11B
CNS: Central nervous system
CPP: Chronic pelvic pain
DAB: 3′3-Diaminobenzidine
EDTA: Ethylenediaminetetraacetic acid
GFAP: Glial fibrillary acidic protein
H&E: Hematoxylin and eosin
H_2_O_2_: Hydrogen peroxide
H-DAB: Hematoxylin-3′3-diaminobenzidine
I.P.: Intraperitoneal
IBA1: Ionized calcium-binding adapter molecule-1
IGF-1: Insulin like growth factor 1
IL-1β: Interleukin 1 beta
IL6: Interleukin-6
IU: International unit
L1: Lumbar vertebra 1
NDPI: NanoZoomer digital pathology software
NGF: Nerve growth factor
NLRP3: NOD-, LRR- and pyrin domain-containing protein 3
NMDAR: N-Methyl-D-aspartate receptor
NT3: Neurotrophin-3
NTF3: Neurotrophin-3
PBS: Phosphate buffered saline
PBST: Phosphate buffered saline + Tween 20
PGE2: Prostaglandin E2
PMSG: Pregnant mare serum gonadotrophin
RT: Room temperature
S.C.: Subcutaneous
S100-beta: S100 calcium-binding protein B
SEM: Standard error of the mean
T13: Thoracic vertebra 13
TNF: Tumor necrosis factor
TRPV-1: Transient receptor potential vanilloid 1
VEGF: Vascular endothelial growth factor
