# Supplementary material for: Endometriosis leads to central nervous system-wide glial activation in a mouse model of endometriosis
Source: J Neuroinflammation. 2023 Mar 6;20:59. doi: 10.1186/s12974-023-02713-0 (PMC9987089; doi:10.1186/s12974-023-02713-0)

Additional Figures

Endometriosis leads to central nervous system wide glial activation in a mouse model of endometriosis.

Shah Tauseef Bashir[1,2], Catherine R. Redden [2], Kishori Raj[2], Rachel B. Arcanjo[2], Sandra Stasiak[2], Quanxi Li[3], Andrew J. Steelman[2], Romana A. Nowak[2*]

[1] Department of Molecular and Integrative Physiology, University of Illinois at Urbana-Champaign.

[2] Department of Animal Sciences, University of Illinois at Urbana-Champaign.

[3]Department of Comparative Biosciences, University of Illinois at Urbana-Champaign.

*Corresponding author: Romana A. Nowak

Department of Animal Sciences.

University of Illinois, 1207 W. Gregory Drive, Room 314 ASL, Urbana, Illinois 61801.

Email address: ranowak@illinois.edu.

Additional Figures and Legends

Figure S1.

Overview of the image analysis process. Original images were exported from the NanoZoomer Digital Pathology Image (NDPI) format as TIFF files. Original images were deconvoluted using Fiji's inbuilt “H-DAB” deconvolution option. Finally, we trained (machine learning) the “Trainable Weka Segmentation” plugin to convert the deconvoluted into the soma, filaments, and background. Soma-only segmented images were then thresholded, and soma size was measured using “Analyze particle function” in Fiji with a size threshold of 30 - infinity and circularity threshold of 0.2 - infinity.

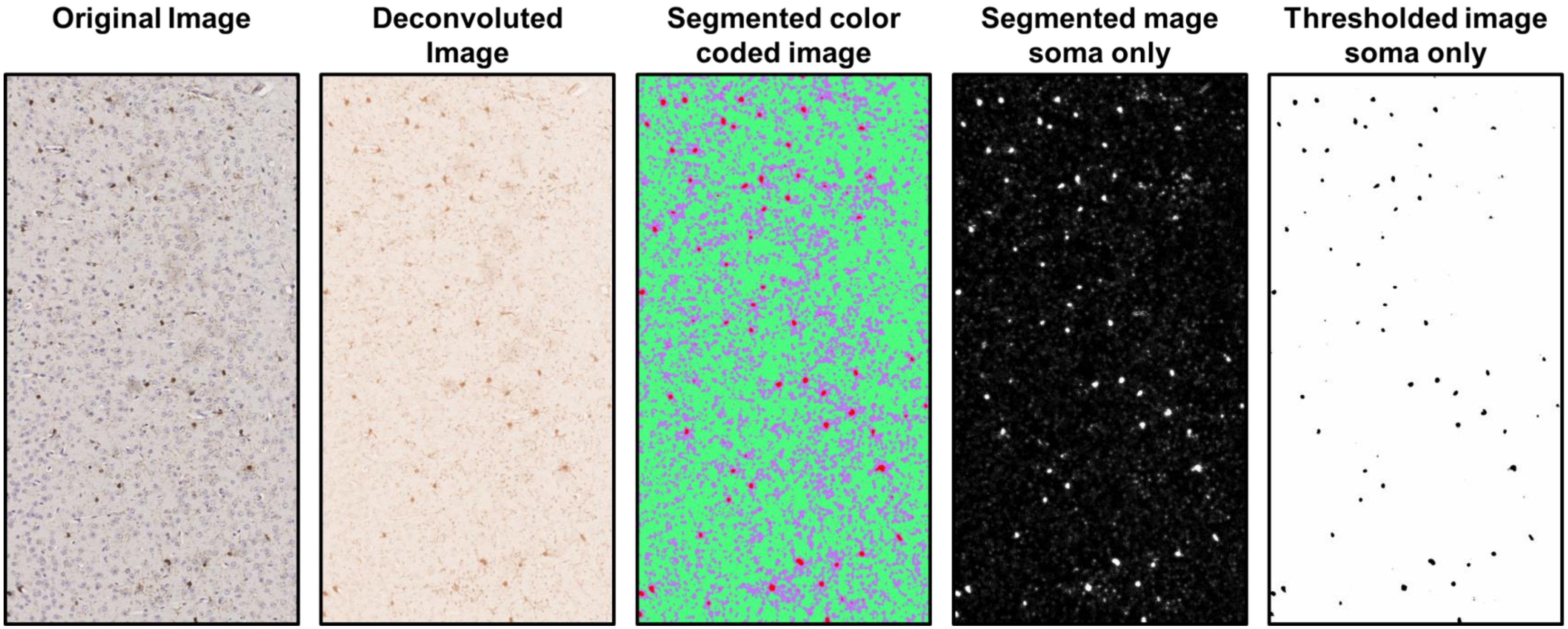

Figure S2.

S100 Beta (S100B) Immunostaining in Various Brain Regions of Sham Control and Endometriotic Mice. A) Representative immunohistochemistry images of S100B in sham controls and endometriotic mice on day 16 and B) day 32, the black bar in the lower right image is equal to 150 and 50 µm for the images and the insets, respectively. C, D, E. F) No difference in the number of astrocytes was observed in the cortex, hippocampus, thalamus, and hypothalamus at days 16 and 32. Values represent mean ± standard error mean (SEM), n=5-6 mice/timepoint.

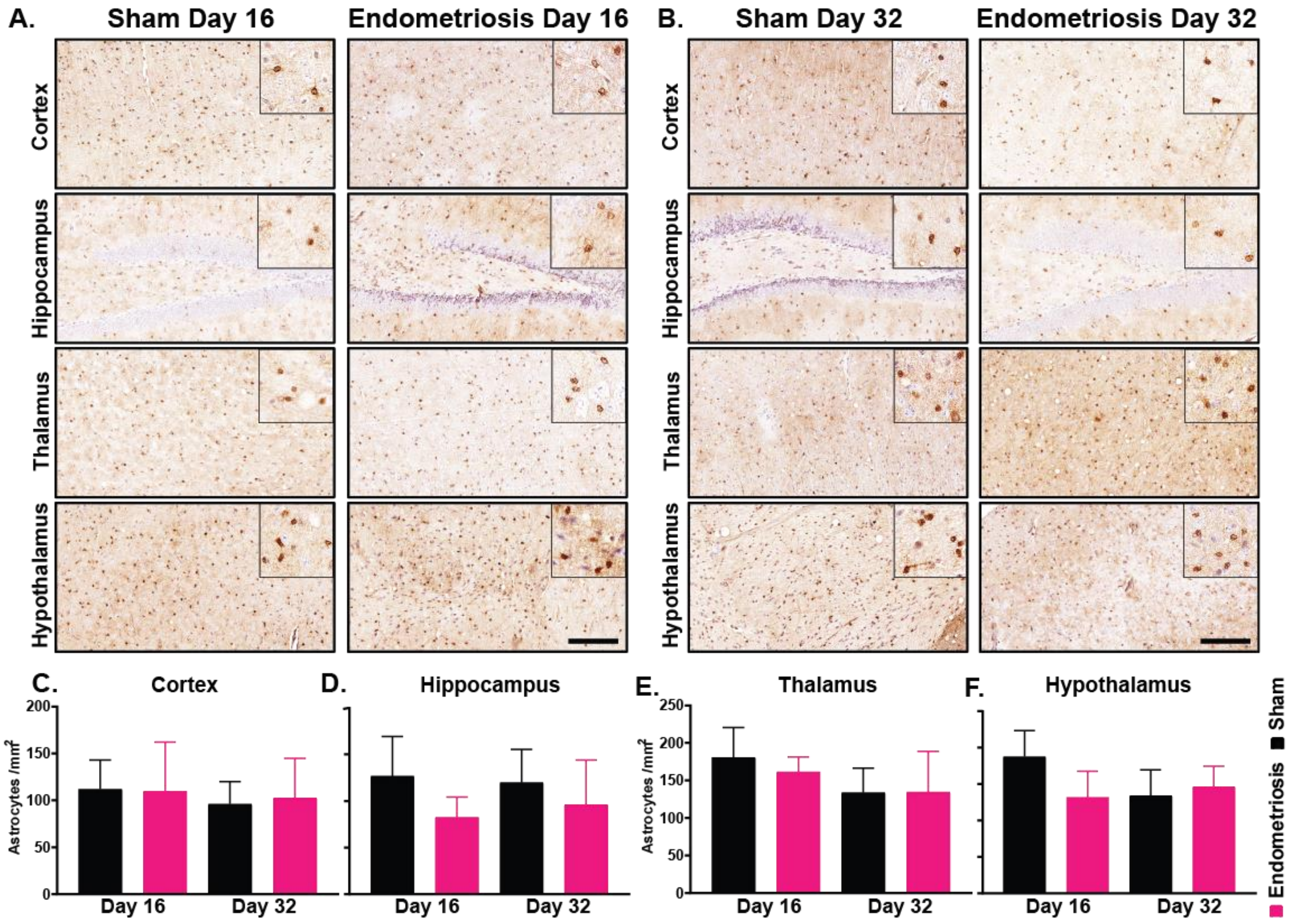

Figure S3.

Heterogeneity in glial number and microglial soma size in sham mice across brain regions. A) The cortex and hippocampus of sham mice had more microglia than the hypothalamus. B) The soma size of microglia in the hippocampus was larger than the microglia in the cortex, thalamus, and hypothalamus. C) The cortex of sham mice had fewer astrocytes than the thalamus and hypothalamus. Values represent mean ± standard error mean (SEM), n=6 mice/timepoint. The asterisks indicate significant differences between groups, ** (p < 0.01), *** (p < 0.001), and **** (p < 0.0001).

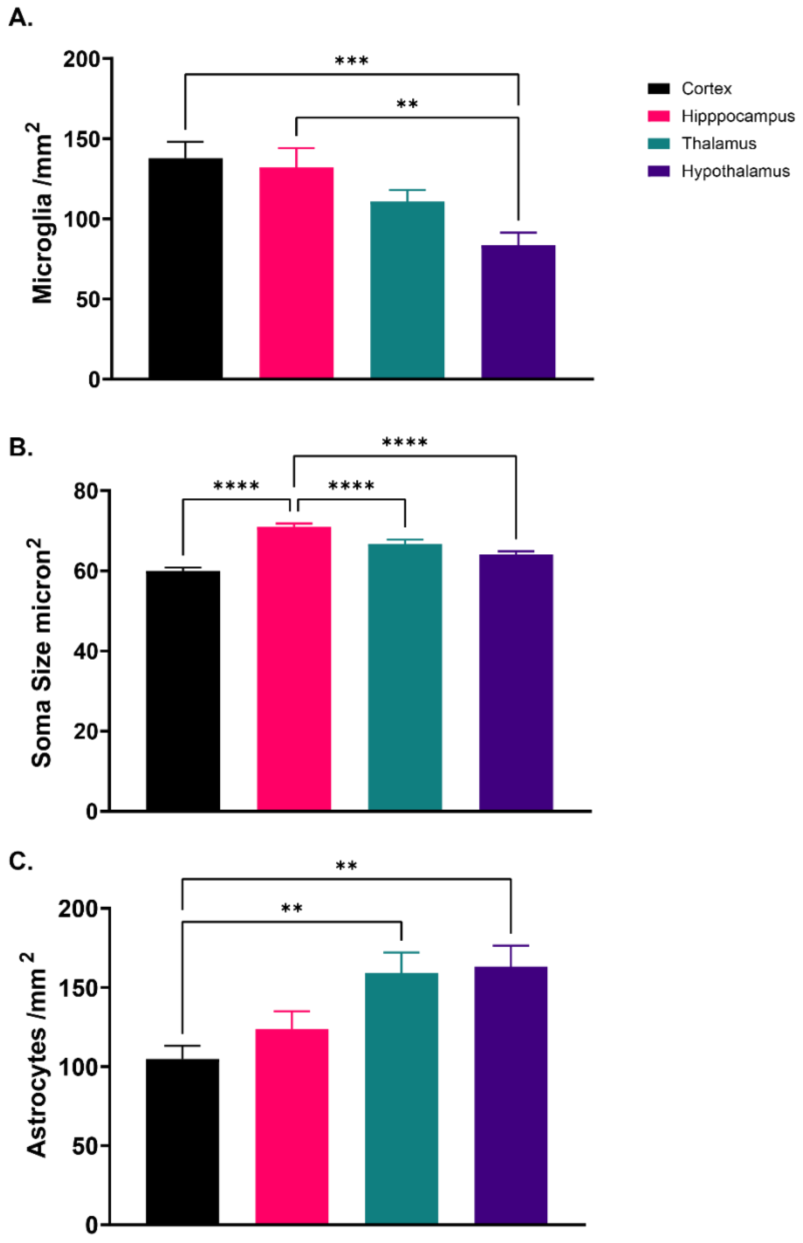

Figure S4.

Heterogeneity in glial number and microglial soma size in mice with endometriosis across brain regions. A) The cortex of mice with endometriosis had more microglia than the thalamus. B) The hippocampus of mice with endometriosis had fewer astrocytes than the thalamus and hypothalamus. Values represent mean ± standard error mean (SEM), n=5-6 mice/timepoint. The asterisks indicate significant differences between groups, * ($p < 0.05$).

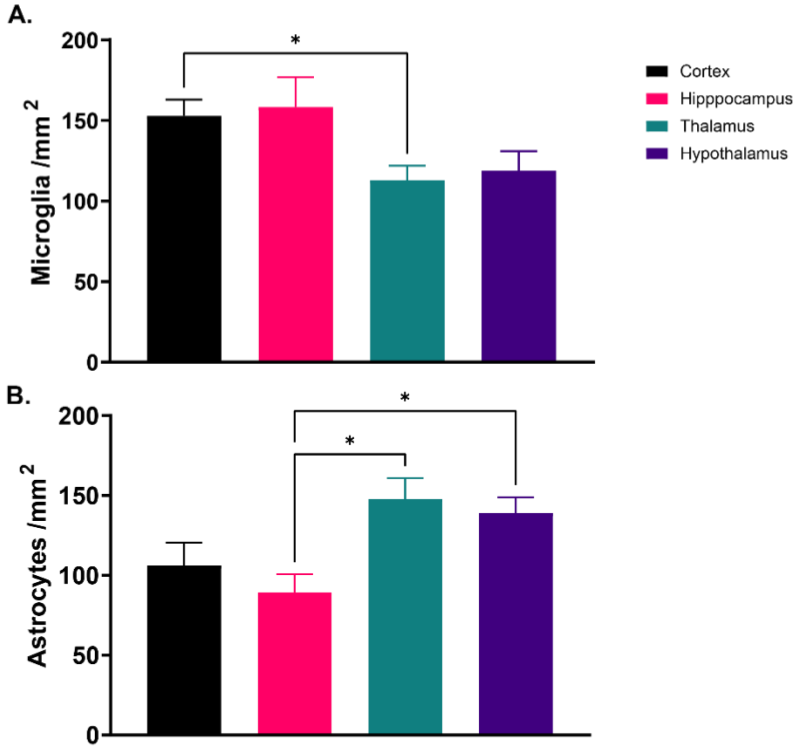

Figure S5.

Expression of TNF and IL6 combined from all brain regions in sham controls and endometriotic mice. A) Mice with endometriosis showed an increased expression of IL6 on day 32 than sham controls. B) Mice with endometriosis showed an increased expression of TNF on day 16 than sham controls. Values represent mean ± standard error mean (SEM), n=5-6 mice/timepoint. The asterisks indicate significant differences between groups, ** ($p < 0.01$).

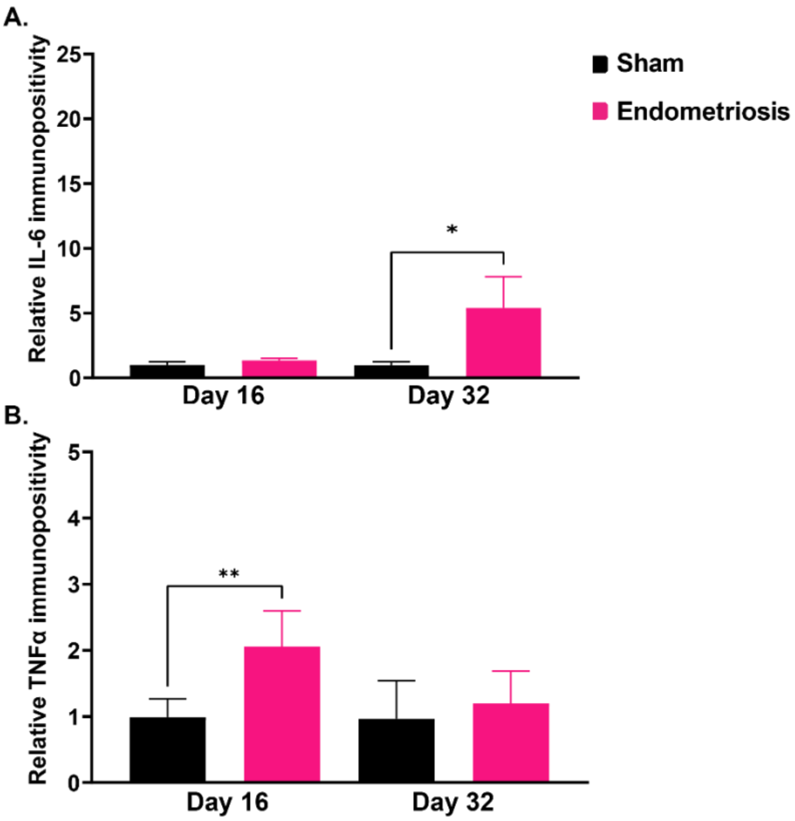

Supplement: Supplementary file 2 — Additional file 2. Additional figures. [file 12974_2023_2713_MOESM2_ESM.pdf]
